# Utilization of additive from waste products with gasoline fuel to operate spark ignition engine

**DOI:** 10.1038/s41598-022-11599-6

**Published:** 2022-05-11

**Authors:** Omar I. Awad, Obed M. Ali, Bo Zhou, Xiao Ma, Ali Thaeer Hammid, Naseer T. Alwan, Salam J. Yaqoob, Saad Motahhir, S. S. Askar, Mohamed Abouhawwash

**Affiliations:** 1 Department of Mechanics and Aerospace Engineering, Southern University of Science and Technology, Shenzhen, China; 2 Renewable Energy Research Unit, Northern Technical University, 36001 Kirkuk, Iraq; 3 State Key Laboratory of Automotive Safety and Energy, Tsinghua University, Beijing, China; 4 Computer Engineering Techniques Department, Faculty of Information Technology, Imam Ja’afar Al-Sadiq University, Baghdad, 10012 Iraq; 5 Technical Engineering College of Kirkuk, Northern Technical University, 36001 Kirkuk, Iraq; 6 Department of Nuclear Power Plants and Renewable Energy Sources, Ural Federal University Named After the First President of Russia B. N. Yeltsin, 19 Mira St., Yekaterinburg, Russia 620002; 7 Department of Research and Education, Authority of the Popular Crowd, Baghdad, 10001 Iraq; 8 Ecole National des Sciences Appliquées, Université Sidi Med Ben Abdellah, Fes, Morocco; 9 Department of Statistics and Operations Research, College of Science, King Saud University, Riyadh, 11451 Saudi Arabia; 10 Department of Mathematics, Faculty of Science, Mansoura University, Mansoura, 35516 Egypt; 11 Department of Computational Mathematics, Science, and Engineering (CMSE), College of Engineering, Michigan State University, East Lansing, MI 48824 USA

**Keywords:** Chemistry, Energy science and technology, Engineering

## Abstract

Impacts of blending fusel oil with gasoline on fuel combustion have been investigated experimentally in the current research to evaluate engine performance improvement and exhaust emission. Tested fuel include F10, F20 (10% and 20% of fusel oil by volume) and pure gasoline as baseline fuel have been used to operate 4-cylinder SI engine at increasing engine speed and constant throttle valve of 45%. The present results reveal a shorter combustion duration and better engine performance with F10 over engine speeds with maximum value of 33.9% for the engine brake thermal efficiency. The lowest BSFC of 251 g/kW h was recorded at 3500 rpm engine speed also with F10. All blended fuel have almost similar COV_IMEP_. Less NO_x_ emission was measured with F10 at 4500 engine speed compared to gasoline. However, CO emissions reduced while higher CO_2_ was observed with introducing fusel oil in the blend. Moreover, HC emission increased an average by 11% over speed range and the highest value was achieved with 10% fusel oil addition compared to 20% and pure gasoline. Accordingly, higher oxygen content of fusel oil and octane number contribute to improve combustion of fuel mixture.

## Introduction

Energy needs are increasing the demands from high population and vast economic development countries like China, India, and Brazil^1–3^. The changing of the environmental conditions, human live needs, and deforestation remains the most significant challenges of the economy in many countries^4–8^. Moreover, plantation is considered as an effective strategy to mitigate the environmental pollution and meet the increasing energy demand as a source of bio-energy production^9,10^. Alcohol fuels for ICE are becoming significant because of decreasing fossil fuel reserves and growing global warming^11,12^. Methanol, butanol, and ethanol are suitable alternatives for fossil fuels as they have various physicochemical properties close to those of gasoline. In general, alcohol fuel is produced from many sources such as biomass that could decrease energy necessity. Many sources in nature can be used for producing alcoholic fuels, like ethanol, most of these resources are renewable including corn, products of sugar cane, barley, and even wastes.

Among the liquid biofuels derived from molasses that is getting acceptance as an internal combustion engine is an ethanol. Fusel oil is obtained from the fermentation process of molasses as a by-product^13^. It is composition depend on the raw source used in molasses production^14^. As mentioned above, the raw materials for fusel oil in Brazil and Turkey are molasses. In other countries, fusel oil can be produced as a by-product from corn, barley. Accordingly, Hiroseet al.^13^ suggested different sources for fusel oils production through the fermentation process.

Alcohol fuels aim to enhance the performance of gasoline engines and decrease their emissions^15–18^. Furthermore, some alcoholic fuels have less price than that of fossil fuel^19,20^. Furthermore, the blending of alcohol with gasoline has a noticeable influence on the properties of the mixture, hence, affecting the performance and emissions of spark ignition engine^17,21,22^. Several alcoholics can be used with gasoline as oxygenated additives to enhance the fuel combustion efficiency especially the high-octane rating alcohols like methanol, ethanol and fusel oil.

Bilgin and Sezer^23^ investigated the engine performance with methanol gasoline blended fuel. They have found the maximum brake means effective pressure with 5% addition of ethanol and 95% gasoline fuel blend. According to Dernotte et al.^24^, the usage of different ratios of butanol with gasoline (Bu 20%, Bu 40%, Bu 60% and Bu 80%) have led to more stable combustion and lower engine cyclic variations. Zaharin et al.^25^ proposed experimentally the addition of isobutanol with gasoline on the engine performance characteristics of 4-cylinders SI engine. Tested fuel samples include pure gasoline, 10% ethanol-gasoline blend, and blend of isobutanol at 5%, 10% and 15% with gasoline. The results reveal higher brake power and lower BSFC with blended fuel which led to improving the engine BTE compared to pure gasoline.

Mourad and Mahmoud^26^ investigated the influence of gasoline with ethanol and butanol blends at 2, 5, 10, 15 and 20% ratio on the engine performance. The results reveal noticeable reduction in fuel consumption by 8.22% under different engine operation conditions. However, engine power reduction up to 11.1% for the fuel blends has been observed. Elfasakhany^27^ found that the maximum engine performance observed with methanol-gasoline and ethanol-gasoline blends compared to other alcohols.

Calam et al.^28^ reported that the engine torque and efficiency enhanced with introducing fusel oil in the blend with gasoline at increasing ratio. Awad et al.^29,30^ evaluated the effect of the fusel oil properties on the fuel combustion characteristics in SI engine. They reported significant improvement in the engine BP and BSFC with reducing the fusel oil water content with shorter combustion durations. Moreover, the lower COV_IMEP_ was obtained under all engine loads for the blend of fusel oil and gasoline.

Fusel oil water content reduction reveals positive impact through enhancing the combustion efficiency, engine performance characteristics and stability using blended fuel. Solmaz^31^ observed a significant impact of introducing fusel oil on the fuel combustion efficiency. Calam et al.^32^ studied the engine emissions with fusel oil–gasoline blend. As a result, a reduction in the NOx emission with blends of fusel oil–gasoline with an increase in CO and HC emissions. Similar results were achieved by other researchers when fusel oil–gasoline used^28,31,33–37^. The utilization of waste as a fuel additive is a low-cost valuable option to enhance fuel quality and reduce environmental pollution. In all the studies reviewed above, recently fusel oil has been recognized as a new candidate fuel that demanded more investigation.

This paper aims to characterize engine performance, fuel combustion and emissions at different friction of fusel oil with gasoline blend. Two different fusel oil blending ratios (10% and 20%) have been considered with gasoline in addition to pure gasoline. In addition, Engine performance test has been verified by increase the speed and make the engine load is fixed. Furthermore, the coefficient of variation was investigated based on IMEP as an important indicator for engine stability.

The present research has been organized as follows: “Experimental setup” section presents the experimental setup. “Results and discussion” section introduces the results and discussion. “Conclusions” section presents the conclusion.

## Experimental setup

Blended fuels of pure gasoline (G100) and fusel oil have been used for engine tests. Fusel oil was supplied from a local Turkish company while gasoline fuel (octane 95) was bought from local petrol gas station in Malaysia. The samples of blended fuel prepared by adding 10% and 10% fusel oil ratio with gasoline and denoted as F10 (10% fusel oil with 90% net gasoline by volume) and F20 (20% fusel oil with 80% net gasoline by volume) respectively. Blended fuel stirred for about 20 min to ensure homogenous fuel blend and the fuel properties measured according to ASTM standard procedures as listed in the previous study^29,38,39^.

**Figure 1:**
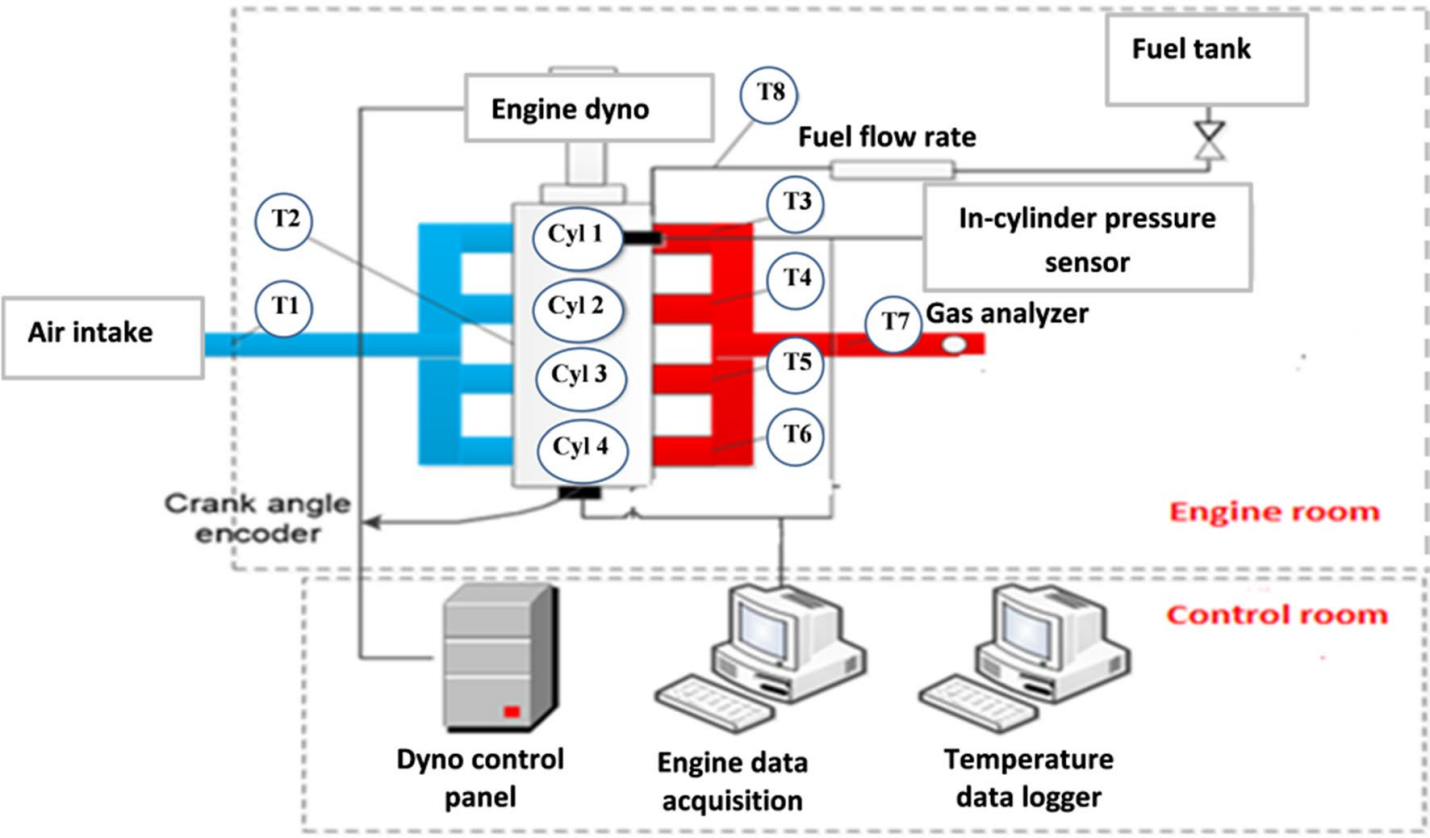
Schematic diagram of the proposed experimental setup.

Naturally aspirated Mitsubishi 4G93 SOHC 4-cylinder PFI gasoline engine was used to perform engine test with the specifications shown in Table 1. Table 2 shows the Exhaust gas analyzer specifications which are used to measure the engine emissions with differently prepared fuel samples. Figure 1 presented the setting of the engine test rig used in this study. Kastler piezoelectric transducer fixed in the cylinder head and used for in-cylinder pressure collection. The specifications of Kastler piezoelectric transducer shown in Table 3. Engine test was conducted under increasing engine speed and constant load of 45% to compare the engine performance and emissions with different fuel samples.

**Table 1:** Engine specifications.

Engine descriptions	Specifications
Number of cylinders	4 in-line
Total displacement (cc)	1834 cc
Cylinder bore (mm)	81.0 mm
Piston stroke (mm)	89.0 mm
Compression ratio	9.5:1
Maximum torque	161Nm @ 4500 rpm
Max power	86 kW @ 5500 rpm
The intake valve opens (BTDC)	14°
The intake valve closes (ABDC)	50°
The exhaust valve opens (BBDC)	58°
The exhaust valve closes (ATDC)	10°
Cooling system	Water cooled
Lubrication system	Pressure feed, full flow filtration
Fuel system	ECI-multi (electronically controlled multi-point fuel injection)

**Table 2:** Exhaust gas analyzer specifications.

Emission	Resolution	Range
NOx	1 ppm	0–5 × 10^3^ ppm
CO	0.01%	0–999 × 10^–2^%
CO2	0.1%	0–16%
HC	1 ppm	0– × 10^3^ ppm
O2	0.01%	0.00–25.00%

**Table 3:** Specifications of Kistler piezoelectric transducer 6125B.

Description	Specifications
Make and model	Kistler ThermoComp 6041A
Range	0–250 bar
Linearity	≤ ± 0.5% FSO
Operation temperature range	− 50 °C to 350 °C
Natural frequency	70 kHz
Sensitivity shift with cooling 50 ± 35 °C	< ± 0.5%

## Results and discussion

The obtained results for the performance of the engine, emissions and fuel combustion have been analyzed and discussed in this section. The tests were conducted with blended gasoline-fusel and net gasoline at 45% engine load and variable engine speed increased from 1500 to 4500 rpm with an increment of 1500 rpm. Engine cyclic variations were analyzed using the coefficient of variations (COV) for 1000 consecutive cycles based on indicated mean effective pressures (IMEP). Moreover, the obtained results were discussed and correlated to the change in the measured properties for different blends shown in Table 4.

**Table 4:** Properties of fusel oil.

Property	Test method	G100	F100	F10	F20
Higher heat value (MJ/kg)	ASTM D 240	43.5	29.93	42.217	40.854
Boiling point (°C)	ASTM D 2887	27–225^40^	98.4	–	–
Moisture content (%)	ASTM D6304	0	13.5	1.35	2.7
Density (kg/m^3^)	ASTM D 4052	769	844	777	785
Research octane number (RON)	ASTM D 2699	95	106	96.1	97.2
Oxygen (%)		0	30.32	3.032	6.064
Carbon (%)	ASTM D5291	87.5	54.2	84.17	80.84
Hydrogen (%)	ASTM D5291	12.5	15.1	12.76	13.02
Sulphur	ASTM D1552	0.1	0.38	0.128	0.156
Kinematic viscosity (mm^2^/s)		0.49	4.1588	0.86048	1.22696

### Engine performance

**Figure 2:**
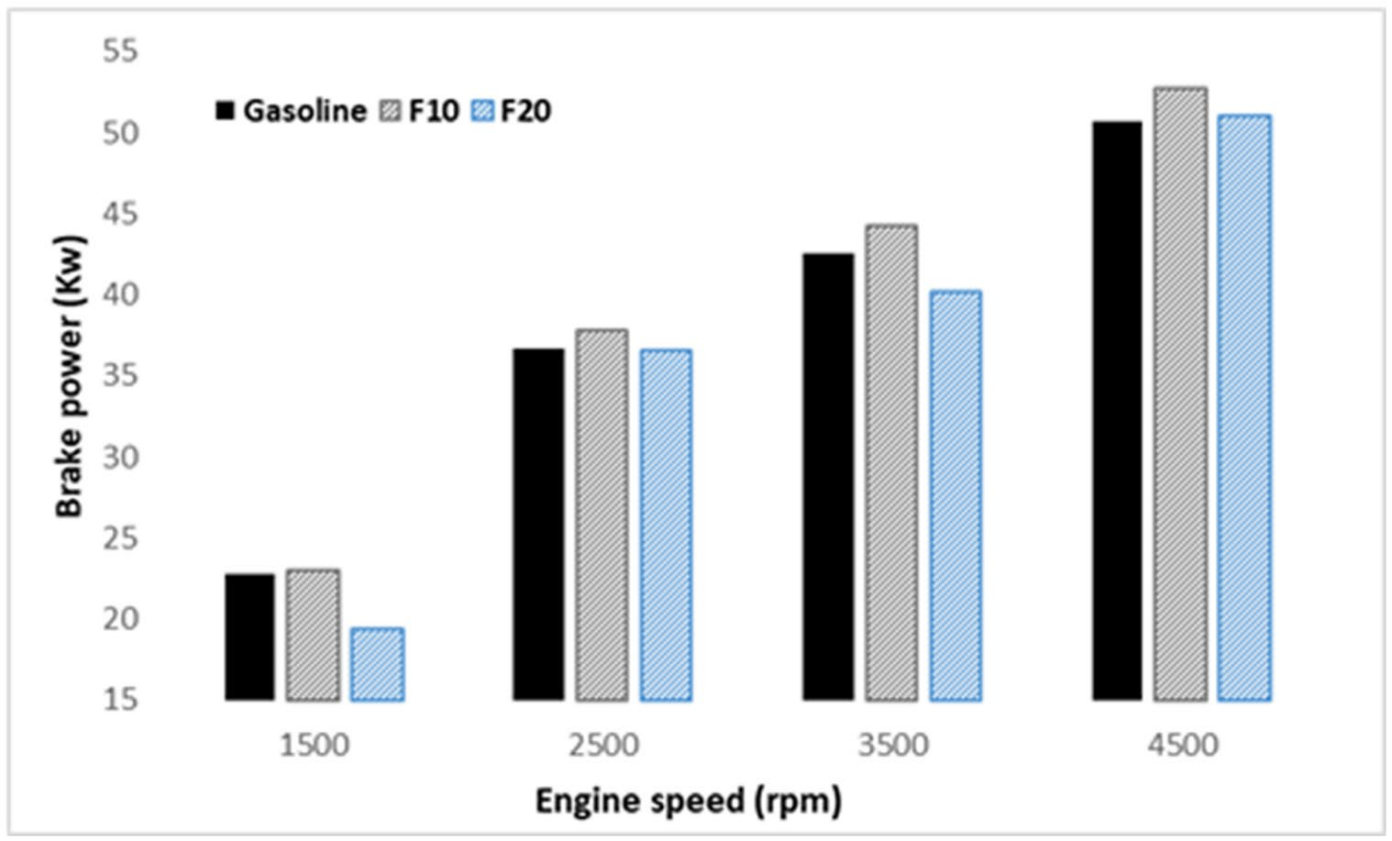
Brake power variation with increasing engine speed.

**Figure 3:**
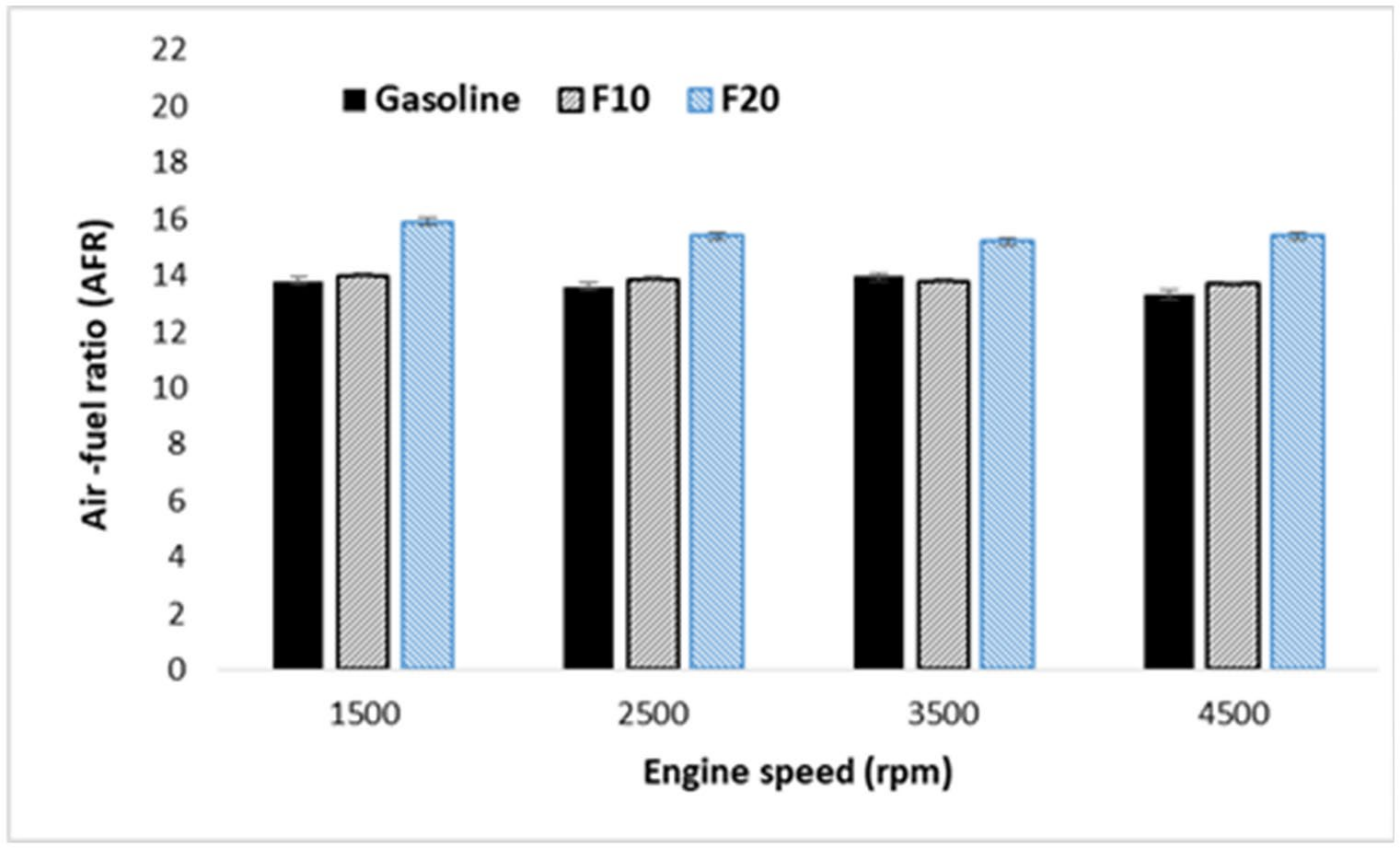
Air–fuel ratio against engine speeds and three different fuel.

Investigation of engine performance operated with the prepared fuel samples is performed using different performance indicators. Figure 2 represented the brake power generated from the engine at an increasing speed. As illustrated, a blend of 10% fusel fuel with gasoline significantly increases the engine BP with increasing engine speed compared to that of pure gasoline. The maximum brake power is observed to be 3% higher for F10 at 4500 rpm engine speed compared to that of pure gasoline. This trend of change can be attributed to the high oxygen concentration and octane number of fusel fuel as presented in Table 4. However, further increase of fusel fuel ratio in the blend to 20% lead to a slight reduction in the brake power than pure gasoline. In this situation, two conflict factors are contributing to this trend of change: fuel energy content, and air–fuel ratio conduction. In general, gasoline has a higher energy content and a lower octane number compared to fusel oil as shown in Table 4. This leads to enhanced fuel combustion and improved engine output power at 10% blending ratio. Further increase of fusel fuel ratio to 20% resulted in a sightly drop in the engine performance it could be explained by the high moisture content of fusel oil as shown in Table 4. The trend of lean and stoichiometric fuel of gasoline -fusel oil blends as shown in fuel as shown in Fig. 3 could be also explained by the brake power behavior.

**Figure 4:**
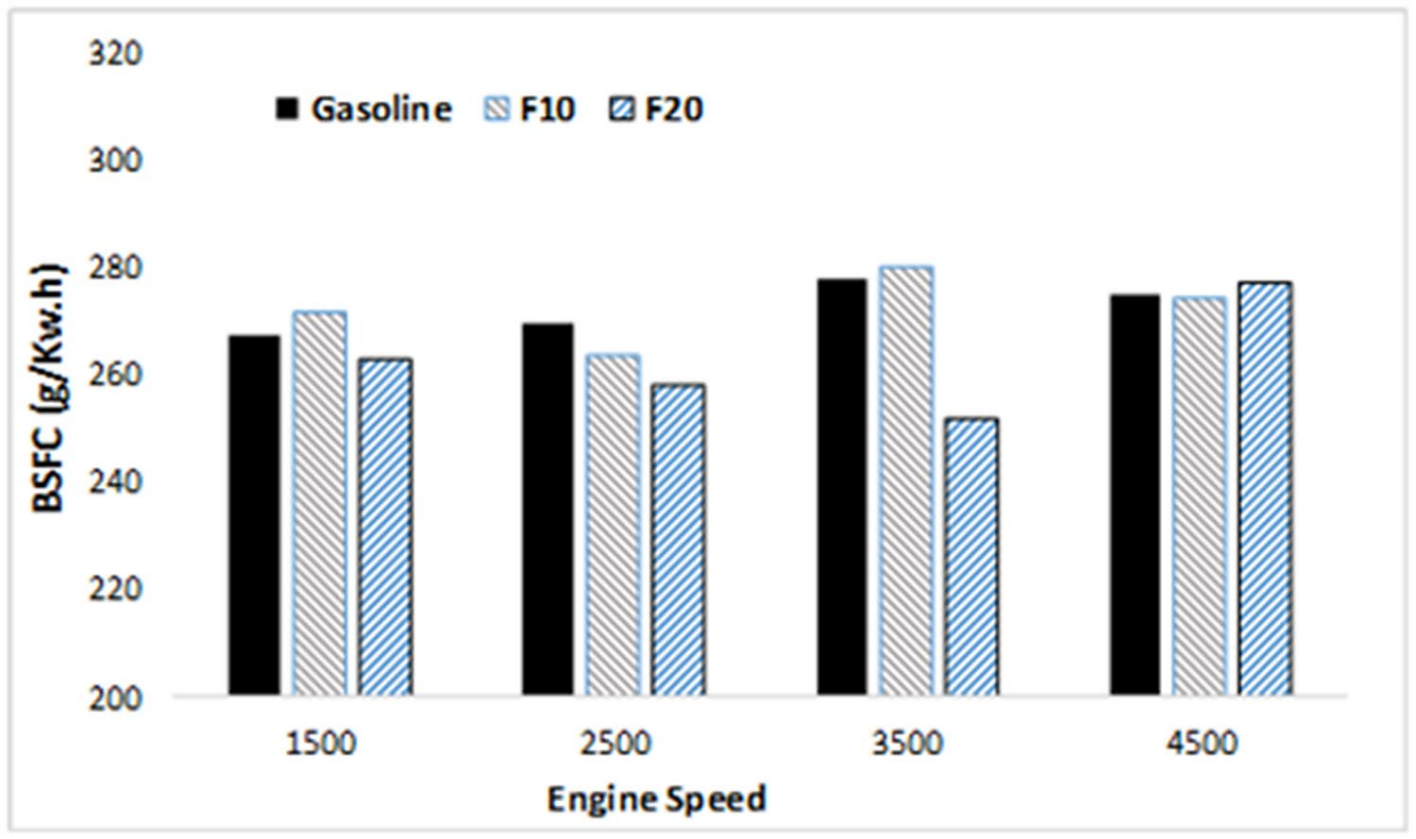
BSFC variation with increasing engine speed.

Engine-specific fuel consumption is another parameter that is used to validate the performance of the engine. It is an important indicator of engine efficiency to produce work with the specific fuel^39^. Figure 4 showed comparable engine-specific fuel consumption at increasing engine speed for all of the tested fuels. However, within the intermediate speed range, increasing fusel oil ratio in the blend drastically reduced the engine-specific fuel consumption. This may attribute to the impact of high octane number and oxygen concentration as shown in Table 4 which enhance the fuel combustion. Furthermore, the BSFC of F10 is higher than F20. Arguably the main reason for the BSFC behavior in this situation is the air–fuel ratio. When F10 runs under 14 air–fuel (rich fuel), more fusel oil will be driven into the piston compared to when F20 runs with 15.4 (lean fuel). Thus, the BSFC decreased by an average of 3%. In this cause two conflicted parameters affecting the engine performance, calorific value and octane number in addition to the fusel fuel oxygen content. When the octane number impact is the dominant, the improvement in the combustion process overcame the effect of the reduction in the calorific value which results in reducing the BSFC.

Brake thermal efficiency (BTE) can be considered as a fuel conversion efficiency indicator with the tested fuel. Accordingly, it can be considered as a more proper parameter than specific fuel consumption to assess the performance of the engine using different fuels. Higher BTE means greater and complete combustion of fuel.

**Figure 5:**
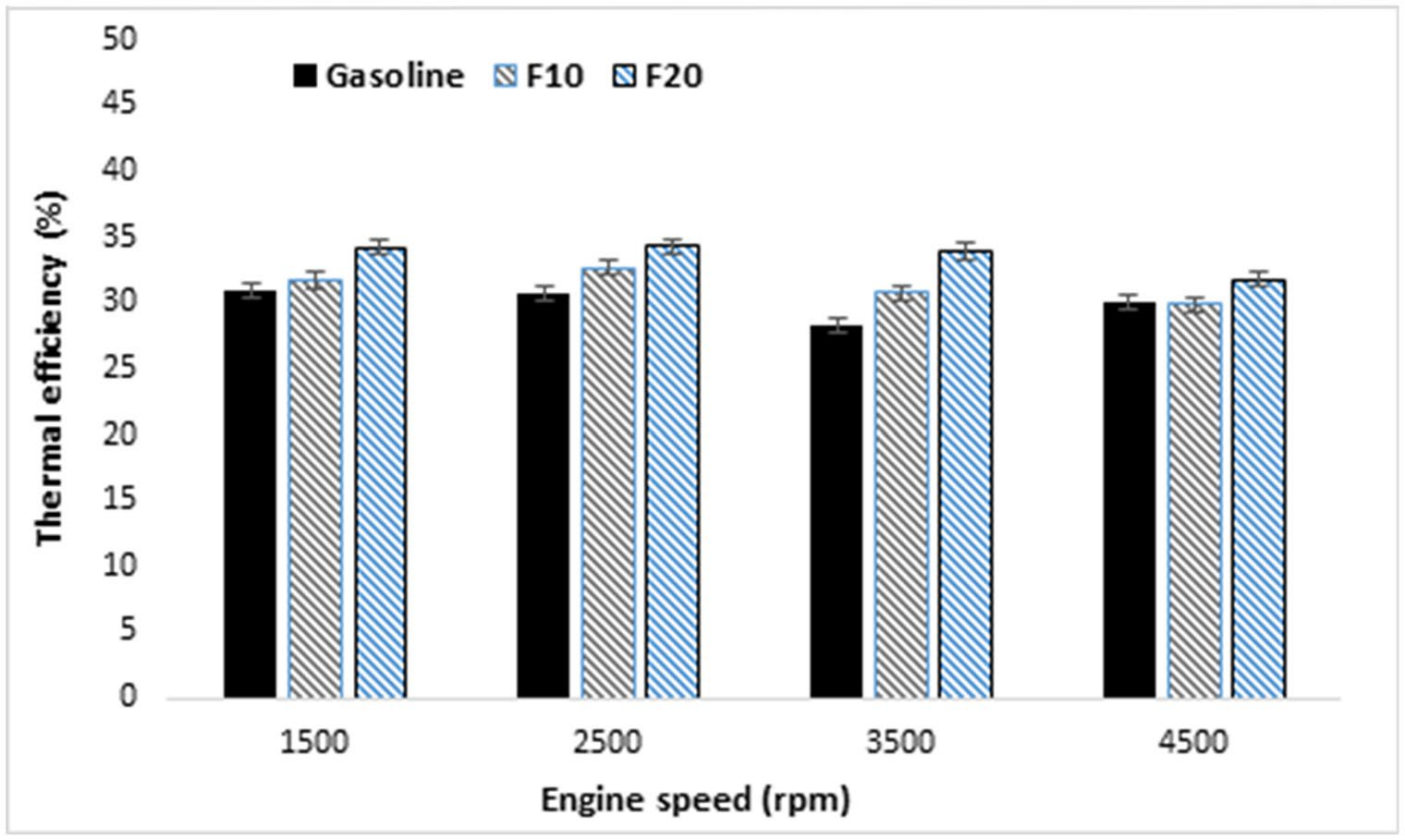
Brake thermal efficiency variation with increasing engine speed.

The variation of BTE and the measurement error bars with increasing engine speed are presented in Fig. 5. It is found that BTE showed a comparable behavior within the adopted engine speeds range. Furthermore, increasing fusel fuel ratio in the blend results in increasing BTE due to the high octane number and oxygen content of fusel fuel which contribute in more complete combustion of the fuel mixture. Furthermore, the enhancement in BTE with increasing fusel oil ratio may be attributed to the increase in reaction activity under rich mixture conditions which results in shorter combustion duration. The maximum engine BTE was 33.9%, at the lowest engine BSFC of 251 g/kW h with F10. The blend of fusel oil–gasoline found to be enhancing the engine brake thermal efficiency.

### In-cylinder pressure analysis

**Figure 6:**
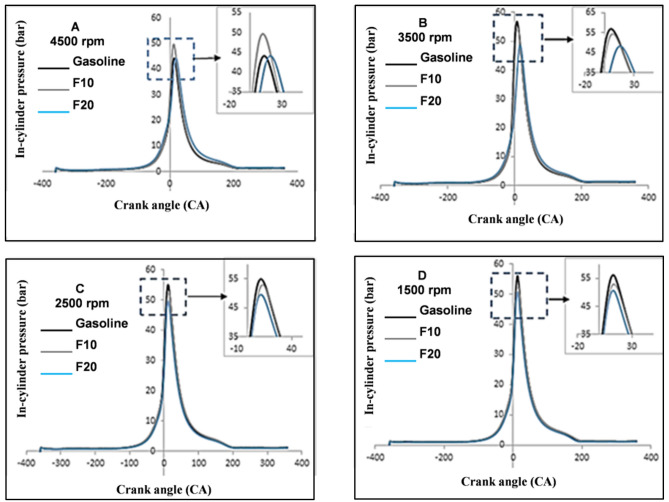
In-cylinder pressure variation with increasing engine speed.

Figure 6 presented the comparisons of the averaged in-cylinder pressures for 1000 consecutive cycles at increasing engine speed and 45% constant engine load. The in-cylinder pressure of F10 was higher compared toF20 and pure gasoline. The main parameters that control this variation in blend fuel are oxygen content, energy content, air–fuel ratio, and the higher latent heat of vaporization for alcoholic fuel. Accordingly, a noticeable improvement was obtained for the in-cylinder pressure under a rich air–fuel ratio with 10% fusel fuel (F10).

The net rate of heat release (ROHR) calculations were obtained from the thermodynamics law based on measured in-cylinder pressure^41,42^. The rate of heat release (dQ/dθ) is calculated based on the following equation:

1 $$\frac{\text{dQ}}{{\text{d}}\uptheta} = \frac{\text{k}}{\text{k}-1}\text{P}\frac{\text{dV}}{{\text{d}}\uptheta} + \frac{1}{\text{k}-1}\text{V}\frac{\text{dP}}{{\text{d}}\uptheta} + \frac{\text{dQth}}{{\text{d}}\uptheta},$$

where the terms of the above equation can be summarized as follows: θ: is the crank angle, k: is the specific heat ratio, V: is the cylinder volume, p: is the in-cylinder pressure.

**Figure 7:**
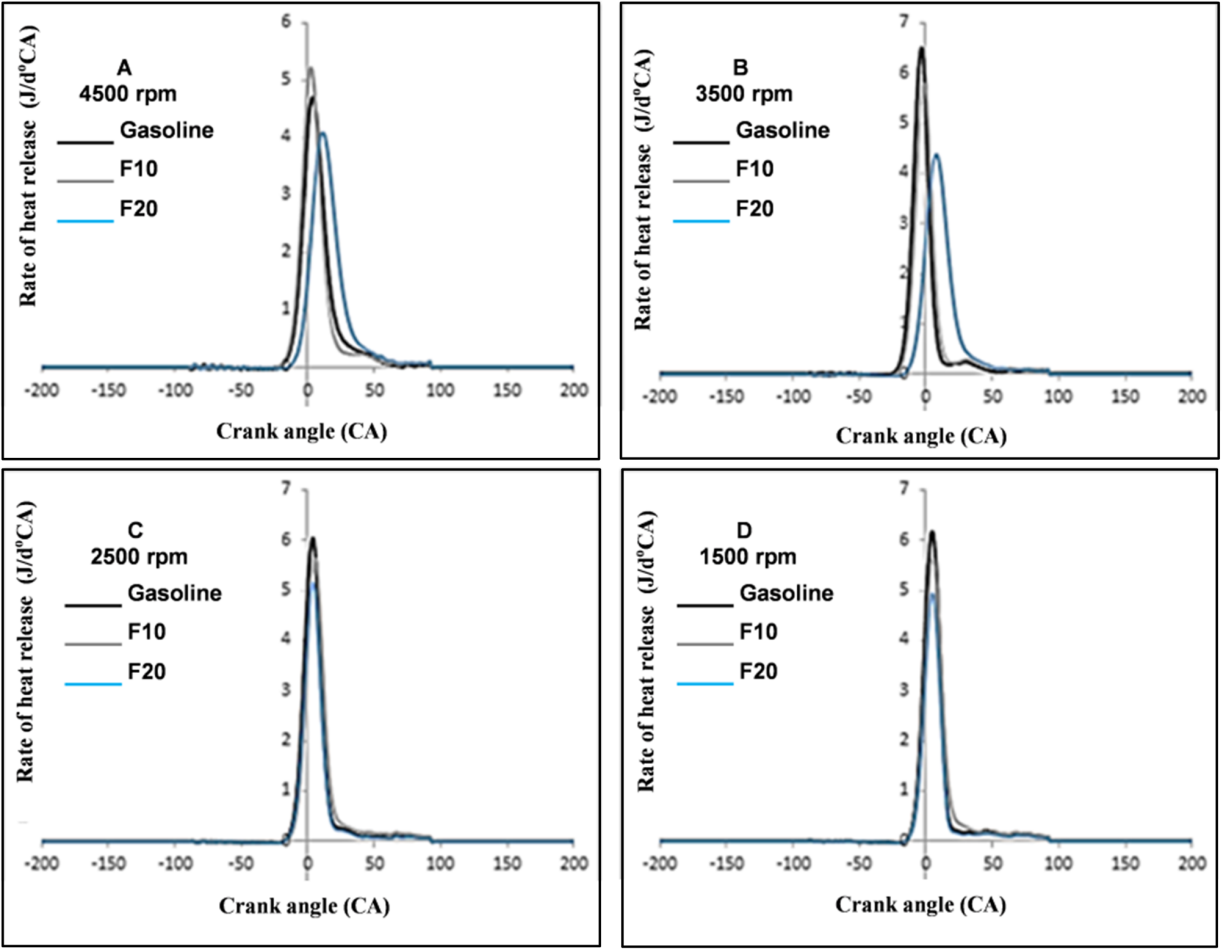
Rate of heat release with increasing engine speed.

Figure 7 showed the differences of ROHR for fusel oil–gasoline blends at 4500 rpm and 45% engine load over 1000 consecutive cycles. It can be recognized from Fig. 7A–D that the ROHR of the F10 and gasoline are almost higher than that of F20. Fundamentally, the ROHR is a reflection of the in-cylinder pressure behavior. A similar result was achieved for the ROHR of all fuel blends.

**Figure 8:**
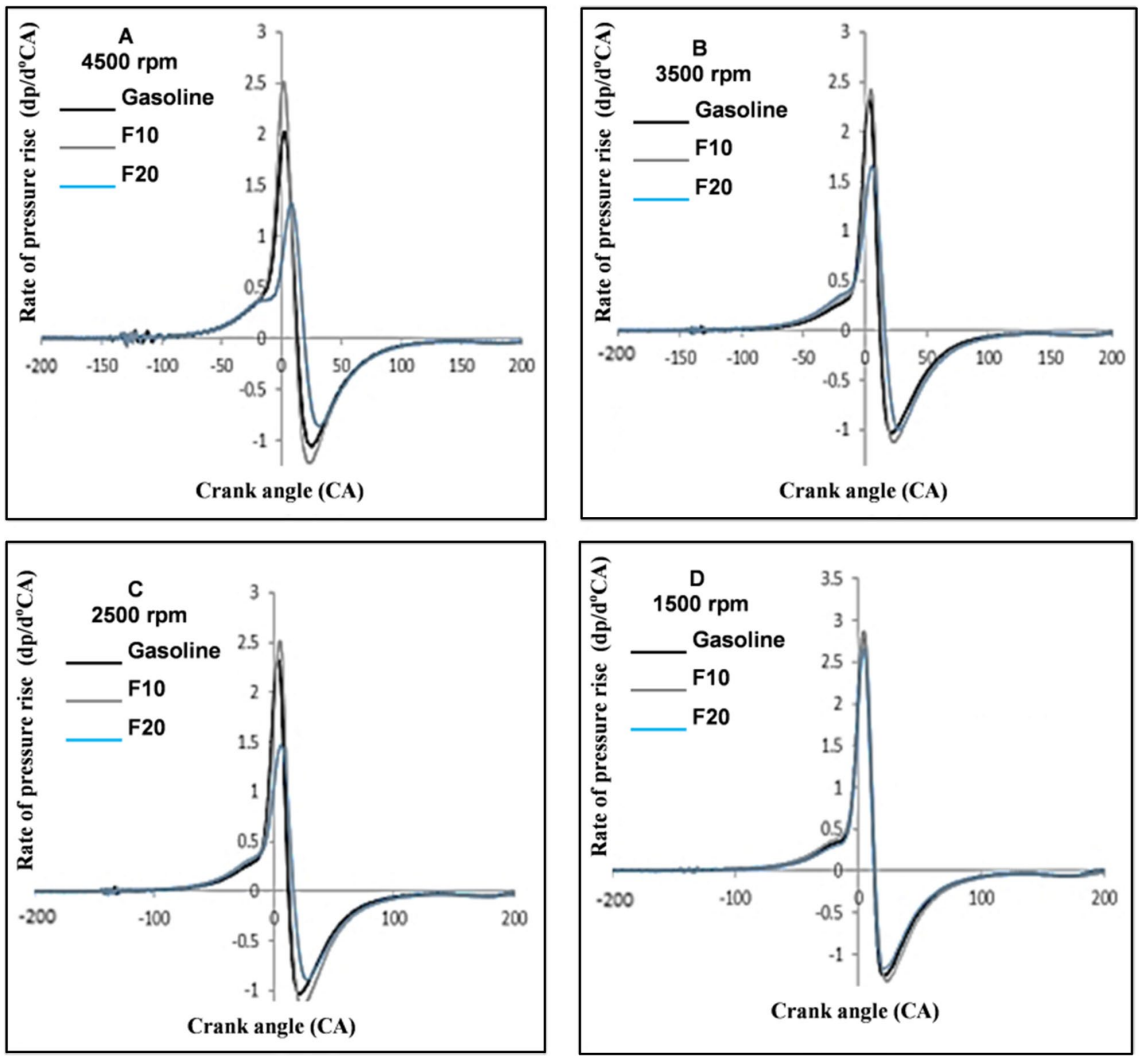
Rate of pressure rise against engine speeds and three different fuel.

ROPR is an important indicator of the energy release rate of the combustion process. It can be computed from the first derivative of the collected pressure. Furthermore, the maximum rate of pressure rise (MROPR) through combustion can lead to unstable operation due to the developed vibration in the crankshaft rotation. If the ROPR value exceeds a limit over 3 bar/CA, this will lead to noisy and rough engine operation. Figure 8 showed the differences of ROPR for blende fuel at 4500 rpm and 45% engine load averaged over 1000 consecutive cycles. The ROPR of the F10 is almost higher than that of F20 and gasoline.

The in-cylinder pressure peak reports the mean effective pressure, and the variation’s coefficient (COV) are considered as significant parameters to be used in the analysis of fuel combustion. Those parameters are being utilized to explain the process of combustion with various fuels. The COVimep is generally utilized to evaluate the cyclic variation in engine^43^ and calculated based on the following equation:

2 $$COV_{IMEP} = \frac{SD(IMEP)}{{\overline{IMEP} }} \times 100,$$

3 $$SD(IMEP) = \sqrt {\frac{{\sum {IMEPi - IMEP} }}{NC}} ,$$

4 $$\overline{IMEP} = \frac{1}{NC}\sum\limits_{I = 1}^{NC} {IMEP} ,$$

where $\text{SD}\left(\text{IMEP}\right)$ is the standard deviation of the IMEP, $\overline{\text{IMEP} }$ is the IMEP mean value and ${\text{N}}_{\text{c}}$ is the number of cycles.

**Figure 9:**
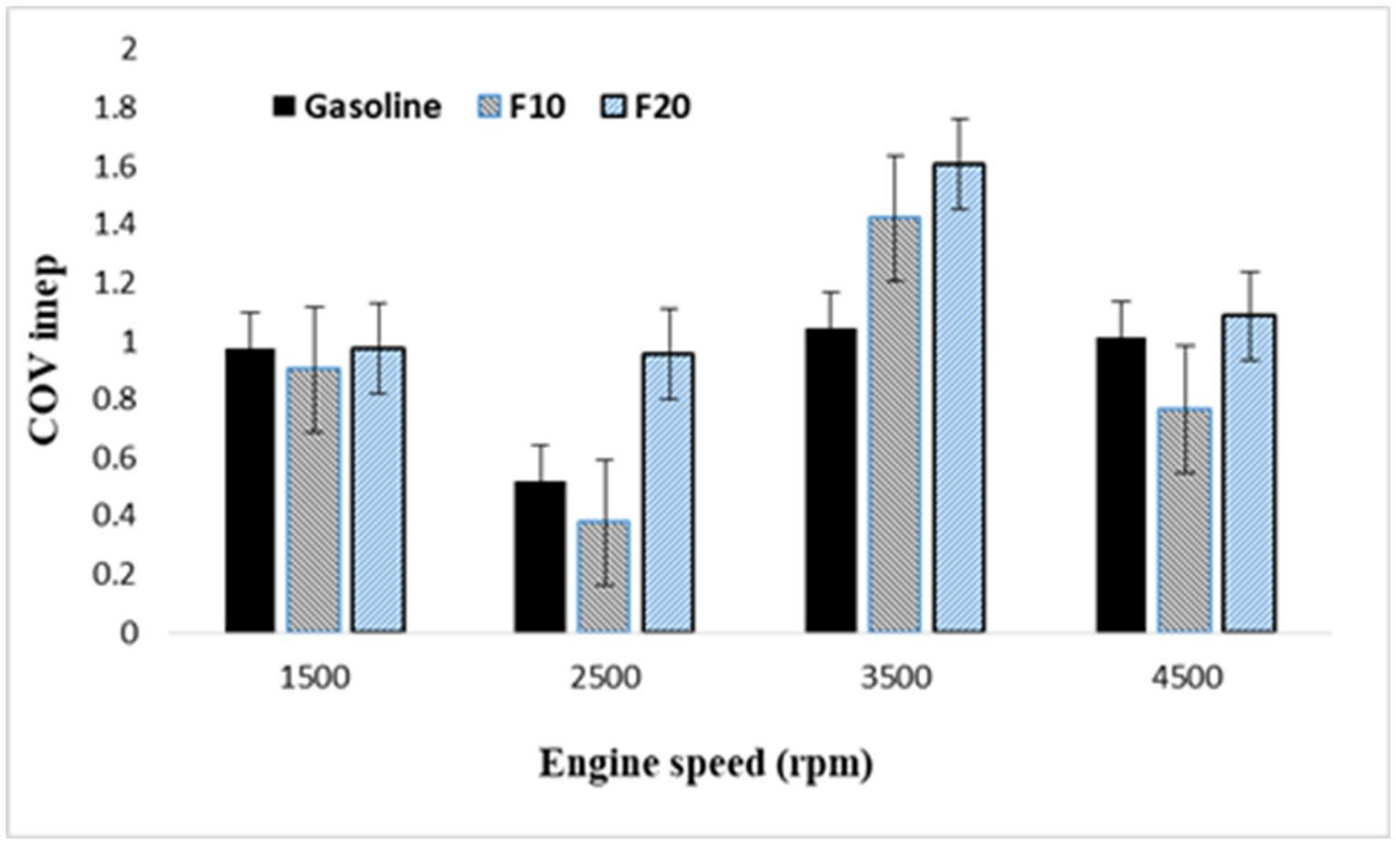
Coefficient of variation of indicated mean effective pressure variation with increasing engine speed.

However, the COV_IMEP_ can be considered as a significant cyclic variability indicator as it bases on pressure data. Figure 9 demonstrates the (COV_IMEP)_ for different fuel blends at 4500 rpm and 45% WOT engine load over 1000 consecutive cycles. The results revealed almost similar COV _IMEP_ with the different fuel samples which means quite stable combustion with these fuels_._ However, the lower COV_IMEP_ obtained with F10 blended fuel under rich air–fuel ratio conditions compared to other fuels.

**Figure 10:**
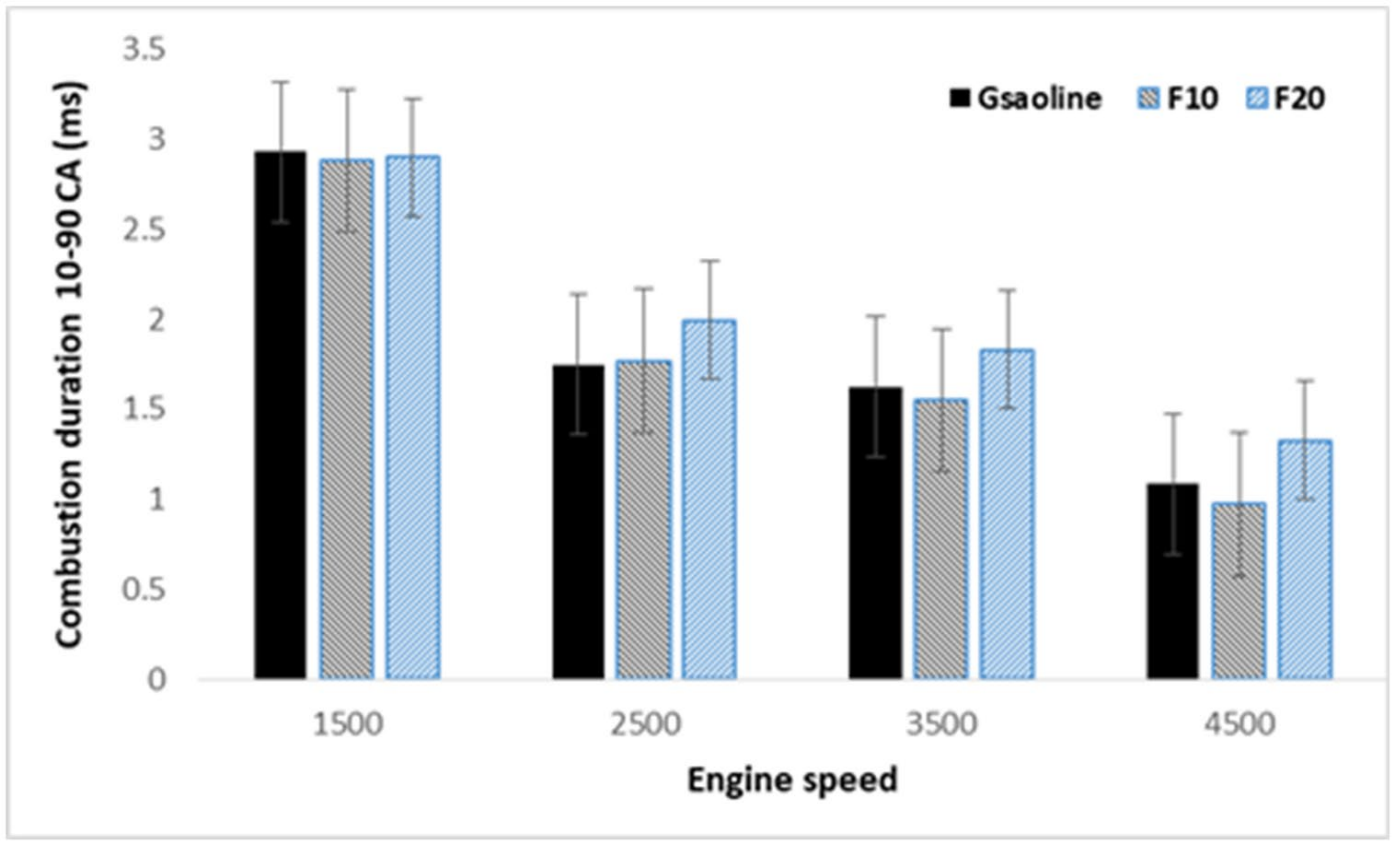
Combustion duration of CA10–90% heat release against engine speeds and three different fuels.

Alcohol permits complete combustion as an oxygenated fuel and provides high volumetric efficiency due to its higher energy content^44^. The fuel type that is utilized in SI engines direct affects the flame speed and flame structure inside the combustion chamber. Durations of flame propagation CA10–90 are important indicators that are influencing fuel combustion and engine thermal efficiency^45,46^. It can be used to determine burning velocity and the efficiency of combustion. In general, the additives of alcohol fuel to gasoline lead to faster burning and shorter flame duration than gasoline. The duration of combustion time is calculated based on crank angle (CA) from the following equation:

5 $$\text{t}\left(\text{ms}\right)=\frac{\text{CA}}{\text{N} \cdot \left(\frac{\text{min}}{60}\right) \cdot \left(\frac{360}{\text{rev}}\right)}\times 1000,$$

where, t and N are the time by millisecond (ms) and engine speed by rpm, respectively. Figure 10 shows the combustion duration of CA10–90% heat release for blended fuel at 4500 rpm and 45% engine load. A shorter duration of combustion is obtained with increasing engine speeds. As a comparison, blended fuel F10 reveals the shorter duration of combustion among the other tested fuels which means higher engine power.

### Engine emission analysis

**Figure 11:**
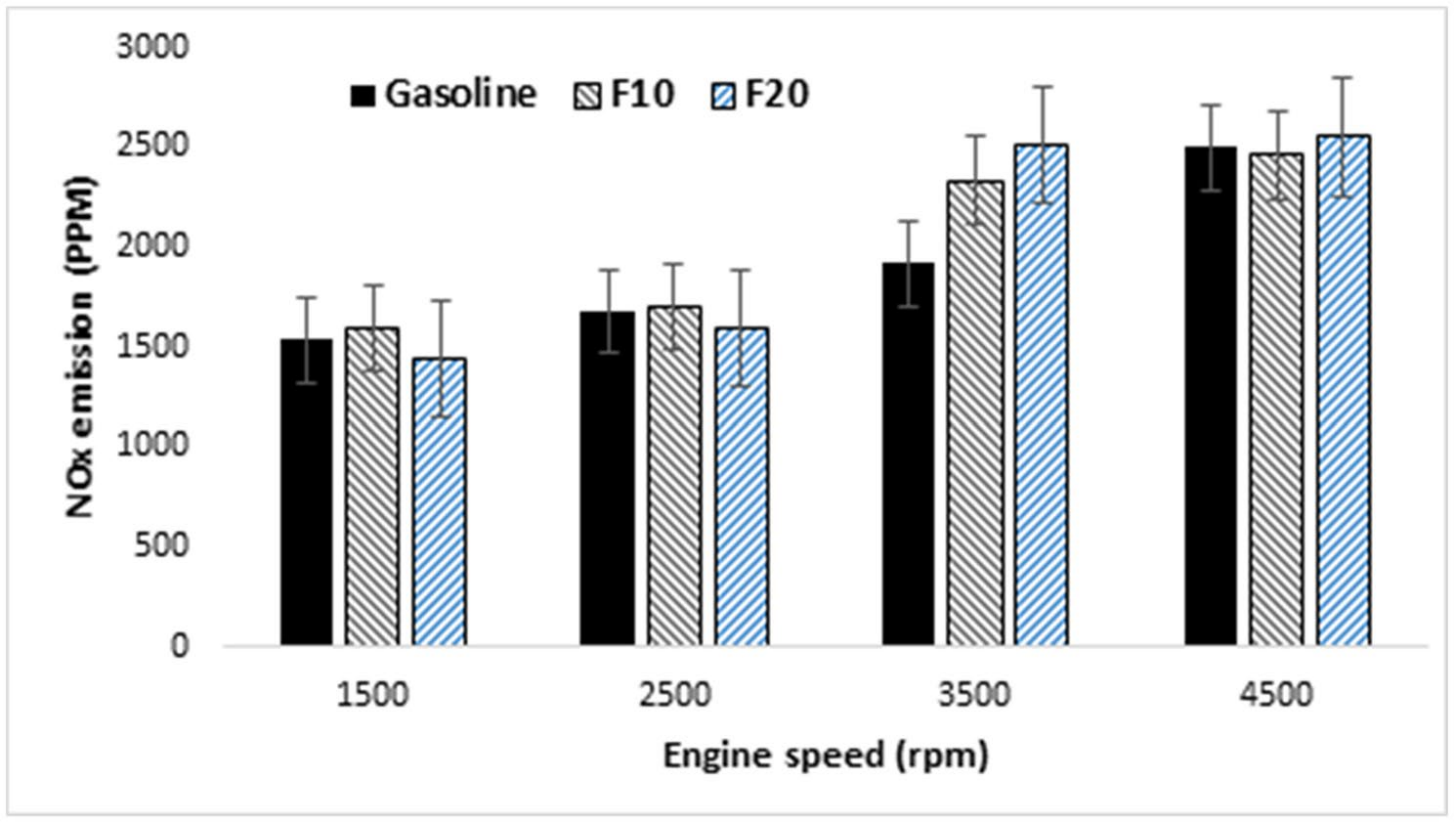
Variation of NOx emissions against engine speeds and three different fuels.

Emitted engine pollution in gasoline engines are related to the fuel properties of air–fuel mixture and operating conditions. Emissions like nitrogen oxides consist of many components which are usually measured together as a NOx. Various literature indicated that, increasing alcohol content in the blend decrease the emission of NOx. The variation of NOx emissions and the measurement error bars in case of increasing engine speed are presented in Fig. 11 for the three tested fuels. In general, NOx emissions are higher for all of these fuels at a higher engine speed as its formation depends on the in-cylinder pressure and temperature at the end of combustion. Furthermore, the concentration of oxygen in the fuel blend also contributes to the formation of NOx emissions at high temperature. At high engine speeds ranging from 3500 to 4500 rpm, blended fuel F20 showed higher NOx emission compared to the other tested fuels and the maximum value is obtained at 3500 rpm. The lowest NOx emission was achieved with F20 at 1500 rpm engine speed.

**Figure 12:**
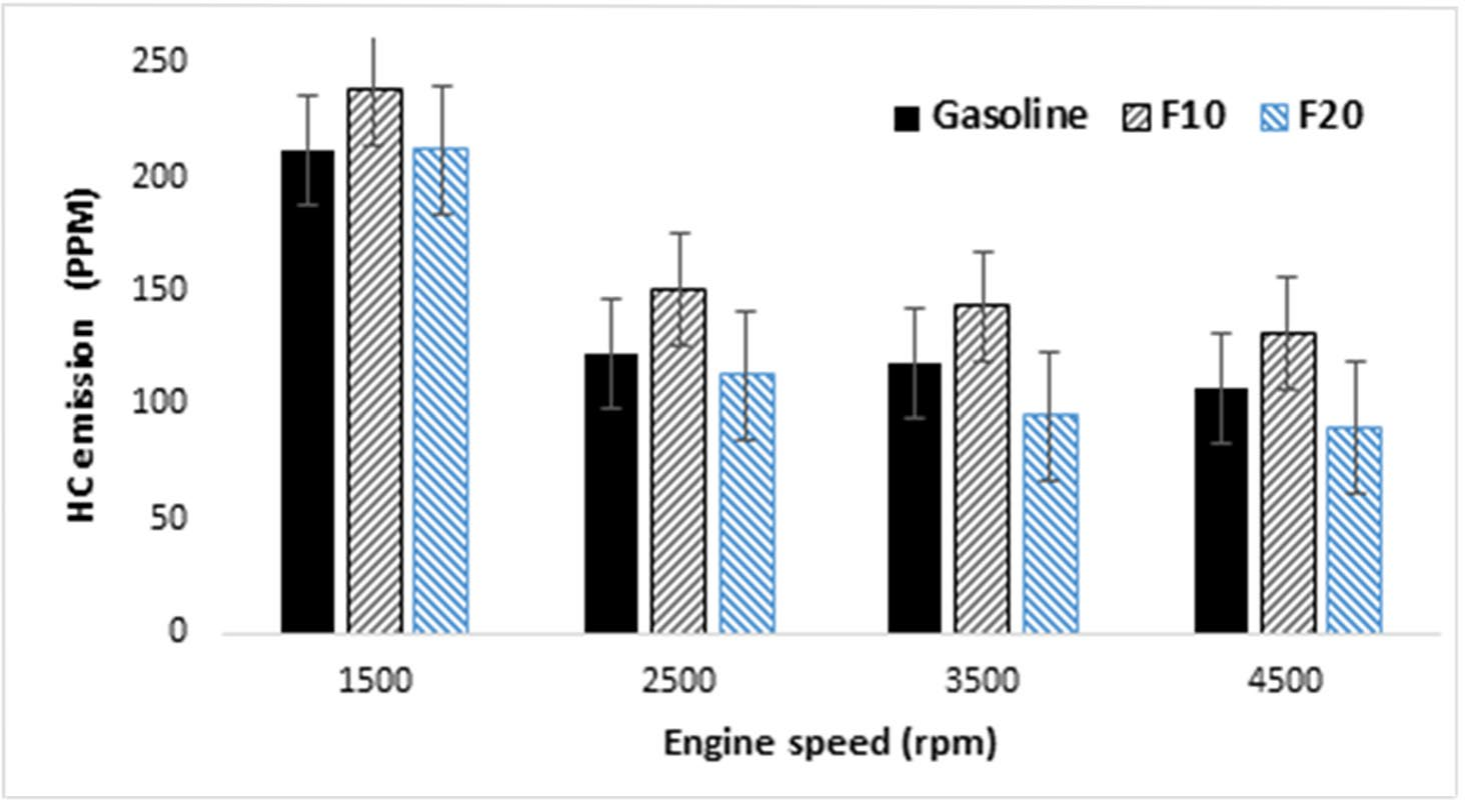
HC emissions variation with increasing engine speeds.

Variation of HC emissions and measurement error bars in case of the increasing engine speed are presented in Fig. 12 for the tested fuel samples. A similar trend of change was observed for HC emission with different fuels with increasing engine speed. As a comparison, blended fuel F10 results in a higher value of HC emission than other fuels with an overall average increase of 11%. Furthermore, HC emissions are decreased for all of the tested fuels at a minimum value for F20 at 4500 rpm engine speed. This is due to the improvement in the mixture homogeneity and reduction of the unburned fuel.

**Figure 13:**
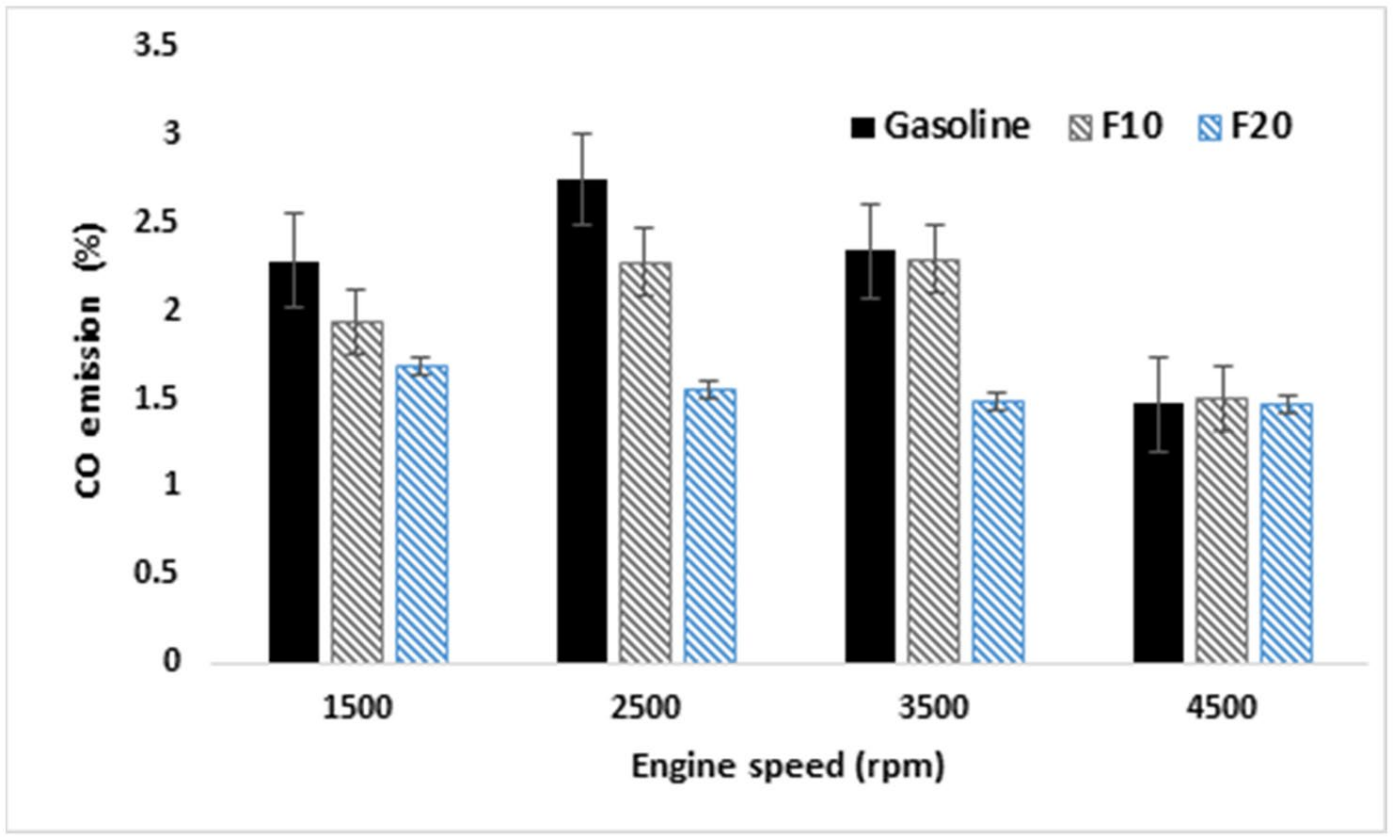
CO emissions variation with increasing engine speed.

CO emissions variation for the tested fuels and measurement error bars are presented in Fig. 13. Comparable behavior was observed for CO emission under low engine speeds from 1500 to 2500 rpm which reduced with increasing fusel oil ratio in the blend within the whole engine speed range. This occurs due to the higher value of oxygen of fusel oil which enhances the combustion of the fuel mixture. Moreover, comparable CO emission is observed at high engine speed, which indicates an improvement in combustion process completion at high engine speed. The CO of F10 is found to be higher than F20 at all engine speeds due to the rich air–fuel ratio of F10.

**Figure 14:**
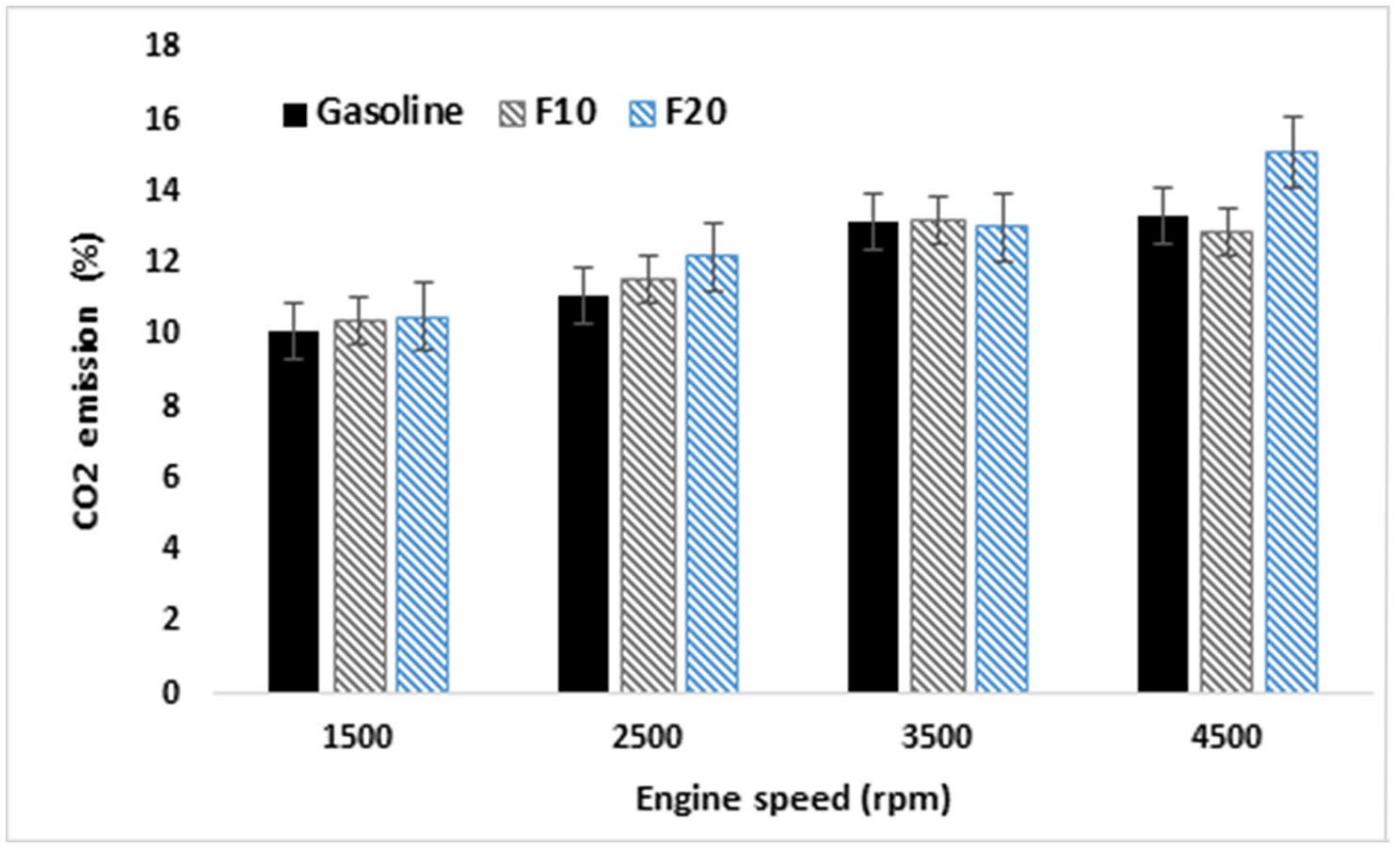
Variation of CO2 emissions against engine speeds and three different fuels.

The variation of CO2 emissions and measurement error bars are presented in Fig. 14 for the tested fuels. Comparable behavior for CO_2_ emission was observes over the low engine speeds ranging from 1500 to 2500 rpm. Further increase in the fusel oil ratio in the blend results in increasing in CO2 emission for the whole engine speed range. Moreover, comparable CO2 emission is observed at a high engine speed of 3500 rpm for all the three tested fuels and the maximum percentage of CO2 is obtained for F20 at 4500 rpm engine speed.

## Conclusions

Experimental tests for the SI engine running with gasoline and fusel oil additive at 10% and 20% ratio and compared the results with pure gasoline fuel were achieved in this study. The engine was running at different engine speeds and 45% WOT.

When compared with gasoline fuel and F20, the brake power and brake thermal efficiency (BTE) of F10 are higher at all engine speeds. Also, the brake specific fuel consumption (BSFC) of F10 was higher than F20 and gasoline. Due to the rich air–fuel ratio with F10, which allows more amount of fusel oil to be driven into the piston compared to the F20, thus, the BSFC increased averagely by 3%. The maximum engine BTE was 33.9%, at the lowest engine BSFC of 251 g/kW h with F10. Additionally, the in-cylinder pressures and rate of heat release (ROHR) are enhanced using the friction of fusel oil at rich fuel under rich air–fuel ratio, thereby, the optimum results were gained with F10.

The obtained results revealed similar COV_IMEP_ with different tested fuels which indicate a quite stable combustion of the fuel mixture. Furthermore, the lower COV_IMEP_ obtained with blended fuel F10 under rich air–fuel mixture compared to other fuels. Shorter combustion duration achieved with increasing engine speeds with the shortest duration observed for blended fuel F10 compared to other fuels. The NOx emission for F10 at 4500 rpm engine speed was lower than that of gasoline. Also, at high engine speeds ranging from 3500 to 4500 rpm, the F20 showed higher NOx emission compared to the other tested fuels and the maximum value is obtained at 3500 rpm. The highest value of HC emission was obtained with F10 compared to other fuels which increased at an average rate of 11% with increasing engine speed. Further increase in CO and CO2 emissions is observed with increasing fusel oil ratio over the whole engine speed.

In a conclusion, the improvement in octane number and oxygen content of the blend fuel results in improving fuel mixture combustion and engine performance under rich air–fuel mixture conditions. As a perspective, further investigation of the functional safety of electrical and/or electronic systems under ISO 26262 and development of a process oriented quality management system under IATF 16949 are essential.

## Data Availability

The data that support the findings of this study are available from the corresponding author upon reasonable request.
